# Genomic comparisons of Persian Kurdish, Persian Arabian and American Thoroughbred horse populations

**DOI:** 10.1371/journal.pone.0247123

**Published:** 2021-02-16

**Authors:** Navid Yousefi-Mashouf, Hassan Mehrabani-Yeganeh, Ardeshir Nejati-Javaremi, Ernest Bailey, Jessica L. Petersen

**Affiliations:** 1 MH Gluck Equine Research Center, University of Kentucky, Lexington, KY, United States of America; 2 Department of Animal Sciences, University of Tehran, Karaj, Iran; 3 Department of Animal Science, University of Nebraska-Lincoln, Lincoln, NE, United States of America; National Cheng Kung University, TAIWAN

## Abstract

The present research aimed to characterize the Persian Kurdish horse population relative to the Persian Arabian and American Thoroughbred populations using genome-wide SNP data. Fifty-eight Kurdish, 38 Persian Arabian and 83 Thoroughbred horses were genotyped across 670,796 markers. After quality control and pruning to eliminate linkage disequilibrium between loci which resulted in 13,554 SNPs in 52 Kurdish, 24 Persian Arabian and 58 Thoroughbred horses, the Kurdish horses were generally distinguished from the Persian Arabian samples by Principal Component Analyses, cluster analyses and calculation of pairwise *F*_ST_. Both Persian breeds were discriminated from the Thoroughbred. Pairwise *F*_ST_ between the two Persian samples (0.013) was significantly greater than zero and several fold less than those found between the Thoroughbred and Kurdish (0.052) or Thoroughbred and Persian Arabian (0.057). Cluster analysis assuming three genetic clusters assigned the Kurdish horse and Thoroughbred to distinct clusters (0.942 in cluster 2 and 0.953 in cluster 3 respectively); the Persian Arabian was not in a distinct cluster (0.519 in cluster 1), demonstrating shared ancestry or recent admixture with the Kurdish breed. Diversity as quantified by expected heterozygosity was the highest in the Kurdish horse (0.342), followed by the Persian Arabian (0.328) and the Thoroughbred (0.326). Analysis of Molecular Variance showed that 4.47% of the genetic variation was present among populations (*P*<0.001). Population-specific inbreeding indices (*F*_IS_) were not significantly different from zero in any of the populations. Analysis of individual inbreeding based on runs of homozygosity using a larger SNP set suggested greater diversity in both the Kurdish and Persian Arabian than in the Thoroughbred. These results have implications for developing conservation strategies to achieve sound breeding goals while maintaining genetic diversity.

## Introduction

The Kurdish horse of Iran is one of the five major Iranian horse breeds. The other Iranian horse breeds include Caspian, Turkoman, Persian Arabian (also known as Assil) and DareShouri. Historical literature chronicles a developmental history of more than 2500 years for Kurdish horses, relating them to an ancient, now-extinct population of horses called “Nesayee.” The Nesayee horses have been documented to have served as transportation for the army of Medes tribe, whose realm was congruent with today’s homeland of Kurdish horses (west of Iran) [1]. It is believed that the Nesayee horses brought to the Southwest of Iran also became the ancestors of the modern Persian Arabian horses.

No formal registry exists for the Kurdish horse, however, the breed is a well-known landrace, selected for agility, dressage gaits, mountain riding and resistance to harsh environmental conditions. We previously characterized the Kurdish horse from a phenotypic perspective by establishing the breed standards, which describe the ideal characteristics used as selection criteria by breeders [2].

The Persian Arabian horse originated in the southwestern part of Iran but currently is a geographically neighboring population to the Kurdish horse and one might suspect some admixture or ancestral relationship between these two populations, as also endorsed by the available historical information. Preliminary genetic studies suggested an immediate common ancestor between Kurdish and Persian Arabian populations [3]. In addition, as these breeds occupy overlapping geographical areas, the question has arisen as to whether they are distinct. Hence, the present research aimed to characterize the diversity of and relationships between these Persian breeds using genome-wide single nucleotide polymorphism (SNP) data from Kurdish horses and Persian Arabians as well as data from the more distantly related American Thoroughbred. We hypothesized that the current population of Kurdish horses can be considered as a unique and homogeneous population distinct from Persian Arabian horses from the genomic standpoint.

## Materials and methods

### Sampled individuals

**Fig 1:**
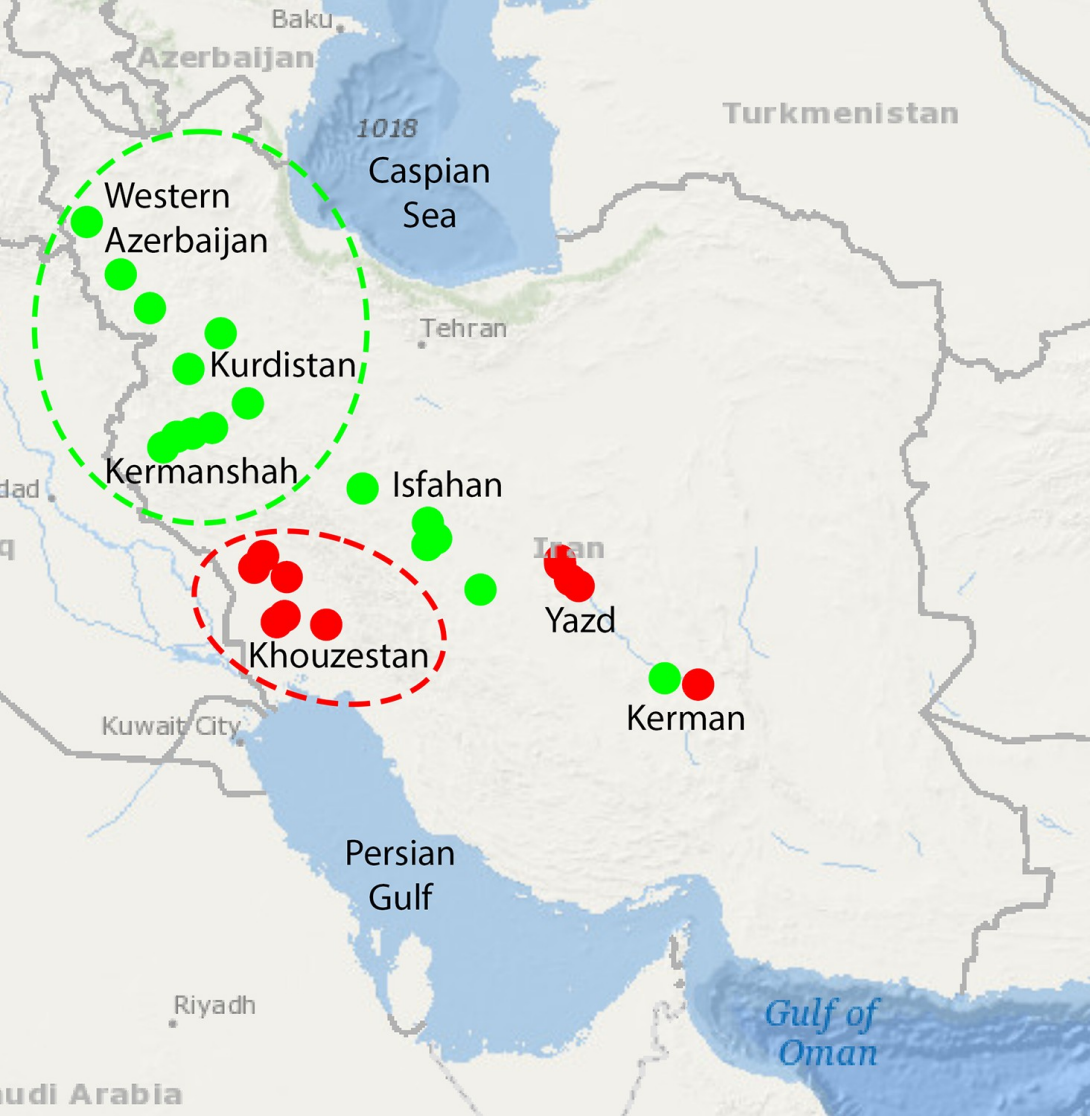
Sampling locations for Iranian populations. Green and red points signify sampling locations for Kurdish and Persian Arabian horses, respectively. The regions circled in green and red identify the original homeland of Kurdish and Persian Arabian horses, respectively. All the point locations outside the ovals represent horses that were descendants of, or were themselves imported from the original homelands. Map imported from the USGS National Map open resources.

We sampled 58 Kurdish horses (43 males and 15 females) distributed over a wide geographic range to ensure the highest level of diversity, including five provinces of Kermanshah, Kurdistan, Western Azerbaijan, Isfahan and Kerman. The 38 Persian Arabian samples (11 males and 27 females), all registered in the Persian Arabian studbook, were obtained in the provinces of Khouzestan, Yazd and Kerman (Fig 1). The Kurdish horses sampled from the central locations (Isfahan and Kerman) did not originate there, rather they (or their ancestors) were imported from the three western provinces (Kermanshah, Kurdistan and Western Azerbaijan). Similarly, the Persian Arabian horses sampled from the central locations (Yazd and Kerman), have their origin from the southwest (Khouzestan). DNA samples were provided from the archive at the University of Kentucky for 83 American Thoroughbred horses (44 males and 39 females). The Thoroughbred horses were randomly sampled from 8 farms in central Kentucky.

### Blood collection and DNA extraction

Blood samples drawn from the jugular vein were collected in 6 ml EDTA vacuum tubes, transported to the lab cooled, and were kept frozen until DNA isolation. DNA extraction was carried out using Phenol-Chloroform protocol [4].

### Ethical statement

The IACUC committee at the University of Kentucky waived review by the ethics committee in 2010 since the Thoroughbred horse samples were obtained from privately owned horses and provided by the owners. Likewise, the samples from Persian horses came from horses that were privately owned and managed, samples were provided by owners so a formal review of the study protocol for ethical treatment of horses was deemed unnecessary. The Department of Animal Science at the University of Tehran is responsible for evaluations of ethical use of animals.

### Genotyping

DNA samples were submitted to Neogen GeneSeek (Lincoln, Nebraska) for genotyping with the Axiom Equine Genotyping Array (Affymetrix Inc.) which harbors 670,796 SNP markers [5].

### Data analysis

SNP & Variation Suite version 8 (Golden Helix, Inc., Bozeman, MT, www.goldenhelix.com) software [6] was used for basic quality control of the genotype data, in which the markers were disqualified if the Minor Allele Frequency (MAF) was < 0.05 and per-SNP Call Rate < 0.95. All the markers on the X chromosome or with unknown genomic location (categorized as CHR_UN) were removed from the dataset. To avoid inclusion of closely-related individuals, the dataset was pruned for Identity by Descent (IBD), such that between each pair of individuals with IBD > 0.2, the one that had more genetic relationship with the other individuals in the sample set was removed. Lastly, SNPs that were in LD across samples were also removed, pruning for an LD threshold of r^2^ = 0.25, considering 100 SNP windows and moving 25 SNPs per set (LD computation method: CHM). As the unequal number of samples among populations could bias LD pruning towards the populations with higher number of samples, we randomly selected 24 individuals from each population and performed LD pruning on the 72-individual subset (24×3), then applied the selected markers to the whole population. To analyze runs of homozygosity, however, LD pruning was not applied although markers with a genotyping rate < 0.95 and the loci on the X chromosome or contigs unassigned chromosomes were removed.

Principal Component Analysis was carried out in the SNP & Variation Suite v8 (SVS). Pairwise *F*_ST_ values between breeds were calculated using SVS and Arlequin version 3.5 [7]. Arlequin was also used to obtain expected heterozygosity (*H*_E_) and population-specific inbreeding (*F*_IS_) values as well as to perform an analysis of molecular variance (AMOVA). Average inbreeding coefficients for each population were calculated using SVS v8 and PLINK 1.07 [8]. To calculate runs of homozygosity (ROH) and obtain ROH-based inbreeding coefficients for each individual, the R package detectRuns was used [9] with parameters: windowSize = 15, threshold = 0.1, minSNP = 15, maxOppWindow = 1, maxMissWindow = 1, maxGap = 1000000, minLengthBps = 250000, minDensity = 1/10000. To quantify ROH of various lengths (0 to ≥48Mb), 20 individuals from each sample were randomly selected in three iterations with the mean and standard deviation in counts/class calculated for each sample. Clustering of breeds into genetic groups was examined using the STRUCTURE program version 2.3.4 assuming K values of 1 to 5, replicating the analysis of each K value five times. The STRUCTURE algorithm assumed the admixture model and correlated allele frequencies. Burn-in iterations of 10,000, 25,000, 100,000, 150,000 and 300,000 reps were tested along with different MCMC repetitions of 100,000, 200,000, 250,000 and 600,000 to confirm convergence. Also, to see if the Thoroughbred samples biased the clustering patterns of Kurdish and Persian Arabian groups, we ran STRUCTURE without the Thoroughbred samples at K = 2. To determine the optimal value of K using the Evanno method [10], the online program STRUCTURE Harvester [11] was employed.

## Results

After data pruning, the final dataset included 13,554 SNPs for a total of 134 individuals out of the 180 horses genotyped. The IBD filtering left 50 out of 58 Kurdish, 24 out of 38 Persian Arabian and 58 out of 83 Thoroughbred horses. For ROH, after removing SNPs with genotyping rate < 95%, 262,390 autosomal SNPs were included in the analysis.

### Principal Component Analysis (PCA)

**Fig 2:**
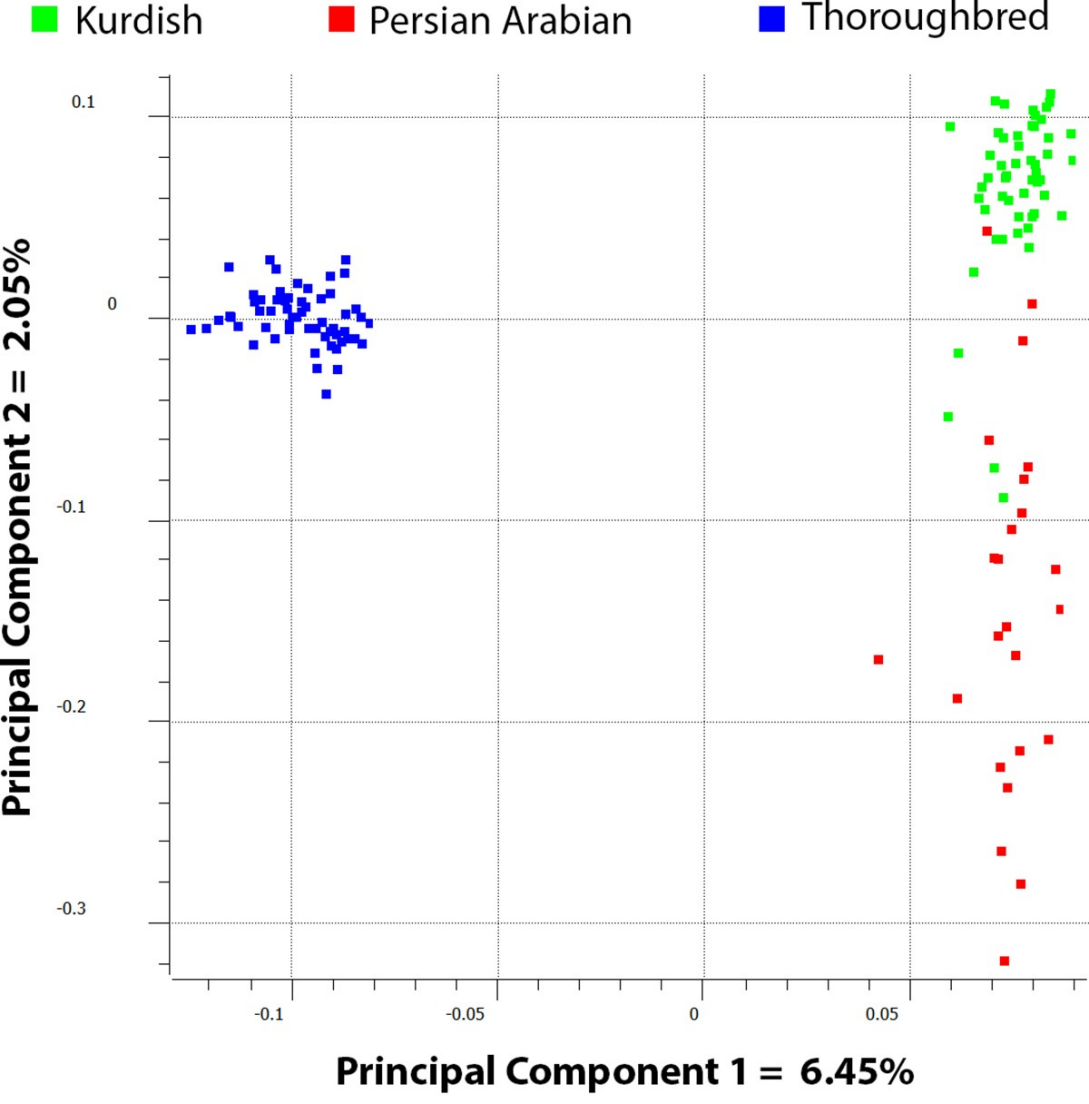
Plot of principal components 1 versus 2 for the 134 horse representing 3 breeds.

The first Principal Component explained 6.45% of the variance, which discriminated the Thoroughbred from both Iranian breeds. The second PC, capturing 2.05% of the variance, showed divergence between the Kurdish and Persian Arabian samples (Fig 2).

The plot also showed that Thoroughbred horses formed a tight cluster. The Kurdish horses clustered in a manner similar to the Thoroughbred, while the Persian Arabian horses had a wider distribution across PC2, where the Kurdish and Persian Arabian clusters were adjacent to one another, with several individuals lying in overlapping regions.

### *F*_ST_

Pairwise *F*_ST_ values were all significantly greater than zero with the least divergence observed between the Kurdish horse and Persian Arabian (Table 1).

**Table 1:** Pairwise *F*_ST_ values between breed groups.

Population Pair	*F*_ST_	*P* Value
Kurdish–Persian Arabian	0.013	<0.0001
Kurdish–Thoroughbred	0.052	<0.0001
Thoroughbred–Persian Arabian	0.057	<0.0001

All of the *P* values were significant (*P*<0.05).

### Population specific inbreeding

Although greatest in the Kurdish horses, no *F*_IS_ value for any sample was significantly greater than zero (Table 2).

**Table 2:** Population-specific *F*_IS_ values.

Population	*F*_IS_	*P* (Rand *F*_IS_ ≥ Obs *F*_IS_)
**Kurdish**	0.005	0.386
**Persian Arabian**	-0.018	0.620
**Thoroughbred**	-0.008	0.636

### Analysis of Molecular Variance (AMOVA)

AMOVA identified 4.47% of the variation present among populations (*P*< 0.001), -0.41% among individuals within populations (*P* = 0.585), and 95.94% within individuals (*P* = 0.065). By eliminating the Thoroughbred from the analysis, the among-population variation remained significant (*P*< 0.001), explaining 1.29% of the variation between the Kurdish and Persian Arabian samples.

### Runs of homozygosity analysis

The Thoroughbred samples had a greater number of longer runs of homozygosity than the Persian and Kurdish horses (S1 Table). The Kurdish horse had fewer ROH ≥ 6Mb than either the Persian Arabian or Thoroughbred, with no ROH longer than 24Mb.

**Fig 3:**
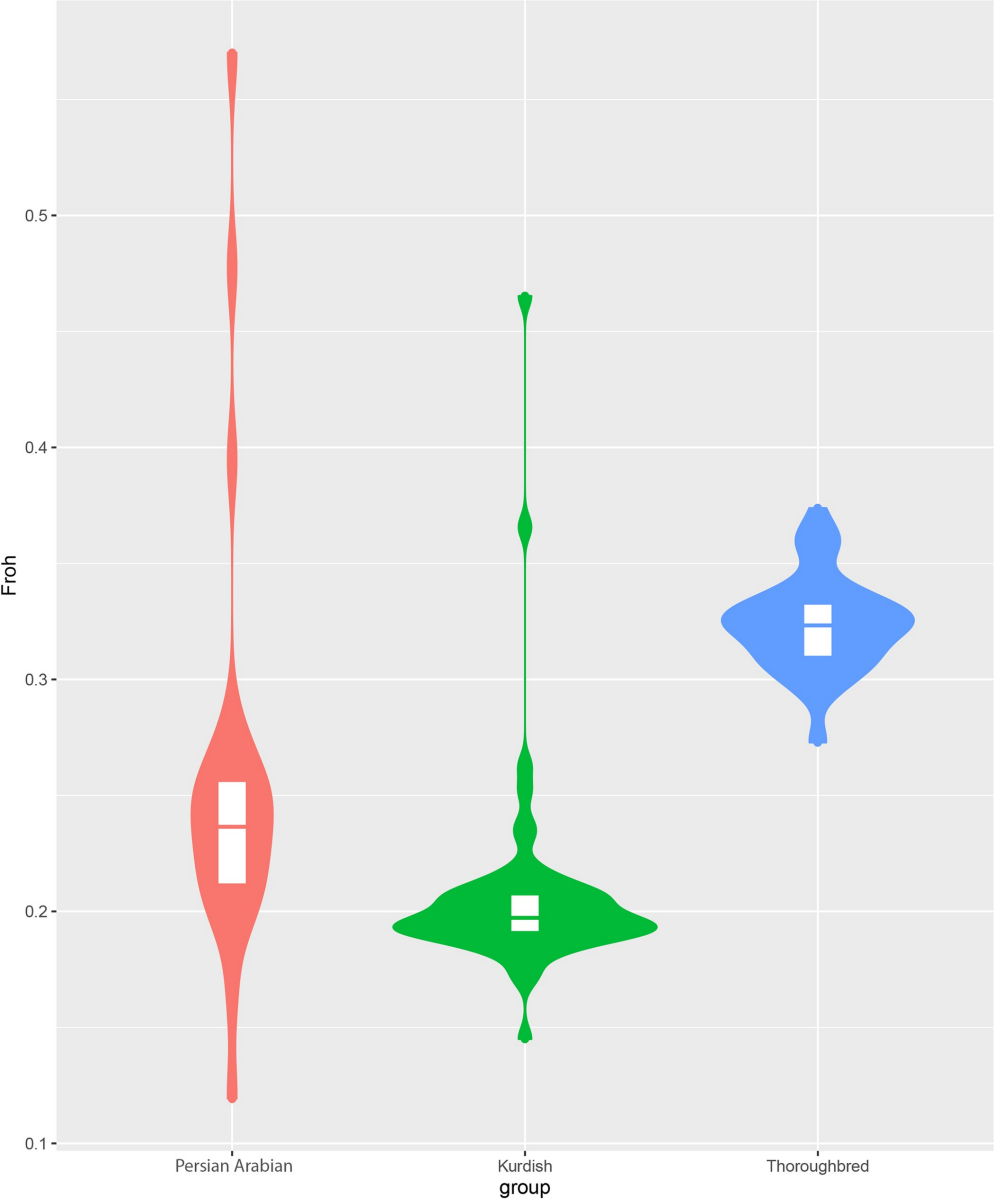
Violin plot of mean, quartiles and the frequency (the width of the plot) of the ROH-based inbreeding coefficient (*F*_ROH_) for each breed group.

The mean individual inbreeding coefficient for the Kurdish horse was less than that of the Persian Arabian (Fig 3), both of which were less than that of the Thoroughbred. Variation among samples was greatest in the Iranian samples, and notably in the Persian Arabian.

The violin plot identifies three outliers in the Persian Arabian group as well as two in the Kurdish population. The same individuals were identified as outliers by PLINK calculations of individual inbreeding. The pedigree of the three outlier Persian Arabian horses available from the studbook confirmed presence of common ancestors in both their paternal and maternal lines and conformed with their higher inbreeding values. For the two outlier Kurdish horses, however, limited pedigree information was available and the extent of shared ancestry could not be determined. Lastly, the correlation between the *F*_ROH_ and the inbreeding values (*F*) calculated in PLINK was 0.765.

### Expected heterozygosity (H_E_)

Expected heterozygosity was greatest in the Kurdish, followed by the Persian Arabian and Thoroughbred (Table 3).

**Table 3:** Average expected heterozygosity values for each breed group.

Population	Expected Heterozygosity
Kurdish	0.342
Persian Arabian	0.328
Thoroughbred	0.326
Total	0.341

### Cluster analysis

For cluster analyses, burn-in and MCMC iterations of 150,000 and 250,000 (respectively) produced consistent results and converged at the highest value of K examined (K = 5). Of the five K values (i.e. the number of subpopulations hypothesized to exist within the entire sample set) tested in this analysis, K = 2 and K = 3 were most informative and were further scrutinized in more detail. Little change was observed when 4 or 5 clusters were considered. The Evanno method identified K = 2 as the best fit for these data (S2 Table).

**Fig 4:**
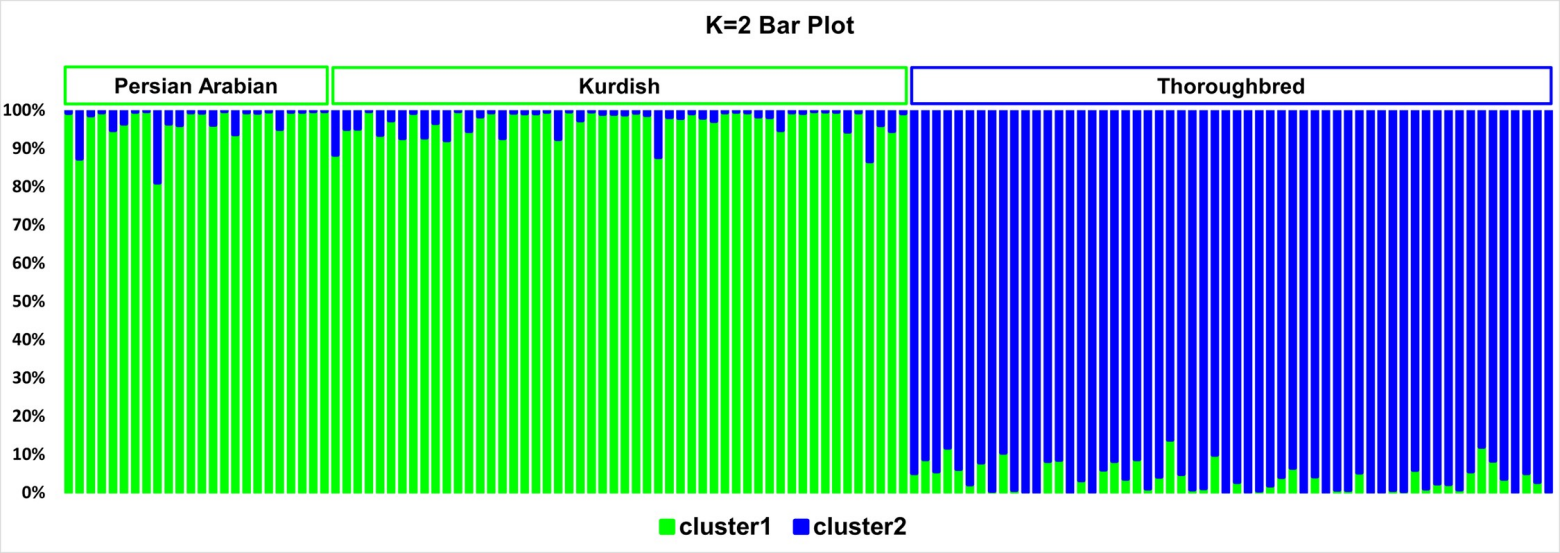
Bar plot of the K = 2 results. The green color designates cluster 1 (which mostly harbored Kurdish and Persian Arabian horses) and the blue color signifies cluster 2 (which mostly contained Thoroughbred horses). Each individual is represented by a single vertical line broken into K colored segments, with lengths proportional to each of the K inferred clusters.

Assuming two clusters, the Thoroughbreds were assigned to a single cluster and the Persian horses (Kurdish + Persian Arabian) to the other (Table 4, Fig 4).

**Table 4:** Average proportion of membership of each pre-defined population in each of the 2 clusters at K = 2.

Population	Cluster 1	Cluster 2
Kurdish	0.971	0.029
Persian Arabian	0.971	0.029
Thoroughbred	0.038	0.962

**Fig 5:**
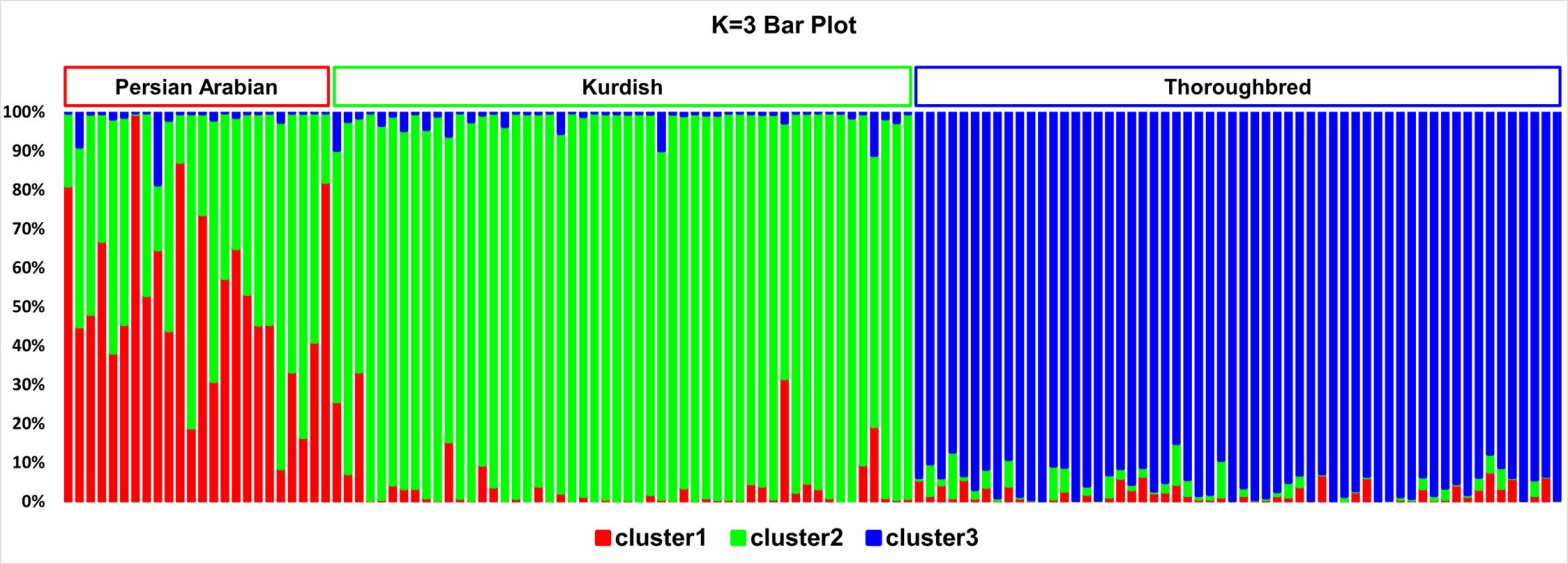
Bar plot of the K = 3 results. The blue color designates cluster 3 (which mostly harbored Thoroughbred horses), the green color signifies cluster 2 (which mostly contained Kurdish horses and covered a part of Persian Arabian’s genome), and the red color represents cluster 1, which is attributable to Persian Arabian. Each individual is represented by a single vertical line broken into K colored segments, with lengths proportional to each of the K inferred clusters.

Assuming three genetic clusters (K = 3), the Kurdish and Thoroughbred horses were each assigned to distinct clusters (Fig 5). The third genetic cluster was found primarily in Persian Arabians, which still showed shared ancestry with the Kurdish horses (S3 Table).

Lastly, running the STRUCTURE program excluding Thoroughbred samples from the dataset (at K = 2) returned similar clustering values for Kurdish and Persian Arabian groups to the original K = 3 results (S4 Table).

For a comparison of outliers/overlapping individuals between Kurdish and Persian Arabian samples, we first identified the individuals in the PC plot that were positioned between the two main breed clusters and ranked them in the order of being closest to the opposing population (based on their PC2 values). Then we examined the individual Structure output for each of the individuals that were intermediate between the two breeds and ranked them in the order of having the highest membership in the opposing cluster. The number and ranking of the outlier/overlapping individuals were identical between the two analyses (6 Persian Arabian and 4 Kurdish individuals). All horses that appear intermediate between the two sets of Persian horse samples were assigned more strongly to the cluster representing the other breed.

## Discussion

Several genetic studies have incorporated horses from Iranian populations [12–19], this is the first, however, to have focused specifically on the Persian Kurdish horse. With respect to our hypothesis, we found evidence that the Kurdish horse population is distinct from but shares ancestry with the Persian Arabians. Additionally, although some individual horses have genomic evidence of inbreeding, overall both Persian breeds appear to be more diverse than the sample of American Thoroughbreds to which they were compared. As our Thoroughbred sample set was limited to a small number of farms in Kentucky, the Thoroughbred analyses in this report may not be generalizable to the whole population of Thoroughbred, but were intended to serve as a comparison to the two Iranian populations. Overall, the results of the present study serve as baseline data characterizing the diversity and genetic make-up of these breeds to allow the development of conservation strategies to achieve sound breeding goals while maintaining genetic diversity of these breeds.

### Analyses of population structure

Our hypothesis that the two Persian breeds are distinct was supported by significant pairwise *F*_ST_ values and AMOVA analyses support the distinction of the Kurdish and Persian Arabian samples. As visualized by the PC plot and cluster analyses, however, there is evidence of gene flow between the two breeds. There are two explanations for this observation. The first is that Persian Arabians might have originated from the Kurdish horse and thus Arabians maintain genomic ties to the Kurdish horses resulting from that founding event, while the Kurdish population has independently evolved. The alternative explanation, considering the Kurdish population may have originated from the Persian Arabian, is based upon the Persian Arabian being a diverse and large breed, relative to the Kurdish horse. A founder effect could have led to the isolation of a portion of this diversity into a new genetic group, that led to, or integrated with Kurdish horses. Gene flow from the Persian Arabian to the Kurdish horse may have resulted from recent population admixture. At the time when the first studbooks for Persian Arabian horses were established around 1976, there was no official breed registry for Kurdish horses; it may be that horse owners with a Kurdish-Persian Arab crossbred or even a Kurdish horse, preferred to have their horses registered with a breed registry, rather than leaving it officially unknown. This would lead to the presence of Kurdish characteristics among Persian Arabian horse populations. Nevertheless, a caveat of cluster analyses and measures of relationships is that directionality of gene flow is not defined; regardless, these data support gene flow between the two Persian populations.

Cluster and PC analyses both suggested that the Kurdish horses are genetically more homogeneous than the Persian Arabians. The observation of higher diversity in the Arabian population as compared to the Thoroughbred is in agreement with reports by Khanshour *et al*. (2013) [12], Sadeghi *et al*. (2018) [15] and is concordant with the latest population study on Arabian horses, which identified a high degree of genetic variation and complex ancestry of those horses from the Middle East, including the Persian group [20]. Diversity of the Persian Arabians in this study is supported by the evidence of gene flow with the Kurdish horse in cluster analysis. Given the observation that the inbreeding at the population level is not significantly different from zero, it may be interpretable that adverse effects of inbreeding depression is less of a concern in the studied populations and there is still a good wealth of genetic diversity which can be used towards breeding if proper management is applied. This is further supported by the analysis of runs of homozygosity. Despite their relatively small population size and likely ascertainment bias of SNP loci favoring detection of rare variants in the Thoroughbred, the Thoroughbred showed a greater proportion of long (>6 Mb) runs of homozygosity, which were less numerous in the Persian Arabian and absent in the Kurdish horse.

### Analyses of individual diversity

With a small population size and lack of formal record keeping, it was of interest to calculate individual estimates of inbreeding based upon both heterozygosity (PLINK) and runs of homozygosity (detectRuns). Although gross calculations of average individual inbreeding present Persian Arabians to be more inbred than Kurdish and Thoroughbred horses, the overall inbreeding level of the Persian Arabian population appears to be driven by the presence of three outliers. Averaging the inbreeding coefficients of the Persian Arabian samples without those outliers, brings the mean inbreeding of the Persian Arabian population lower than that of the Thoroughbred and Kurdish populations. Nevertheless, inbreeding has always been an apparent issue among Persian Arabian horse breeders, as the majority of Persian Arabian lines are descendants of two famous stallions, namely Haddad and Samarghand. Despite considerable inbreeding at the individual level, however, the population has still maintained a good diversity, as implied from their heterozygosity values. Although, their high genetic diversity ensures a healthy population from the genetic standpoint, it is advisable to the breeders to relieve individual inbreeding in their breeding practices, to avoid negative consequences of inbreeding depression. The results also provide another piece of evidence that the Kurdish horse is relatively diverse. This might be due to the fact that the population of Kurdish horses has been mostly shaped by natural selection over a long time rather than human artificial selection.

## Conclusions

■ There is evidence the Kurdish horse population forms a distinct genetic cluster with some individuals showing mixed ancestry.
■ There is evidence of gene flow between the Persian Arabian and Kurdish horses.
■ The overall diversity parameters in the Kurdish horses resembled that of Thoroughbreds. Kurdish horses are a smaller population and it may be appropriate to develop conservation strategies and sound breeding goals to maintain their genetic diversity.

## Supporting information

S1 Table Summary of the total number of runs of homozygosity by each size class. Due to unequal sample size, three iteractions, each of 20 randomly selected individuals was evaluated. Given is the mean and standard deviation (in parenthesis) of the replicates.
(DOCX)

S2 Table Results on the comparisons of K = 1 to 5 tested by STRUCTURE Harvester. The highlighted row belongs to the K value (= 2) that maximizes Delta K per the Evanno method of determining the best fit for the data.
(DOCX)

S3 Table Average proportion of membership of each pre-defined population in each of the 3 clusters at K = 3. (DOCX)

S4 Table Average proportion of membership of each pre-defined population (excluding the Thoroughbred samples from the dataset) in each of the 2 clusters at K = 2. (DOCX)
