# Supplementary material for: Genomic comparisons of Persian Kurdish, Persian Arabian and American Thoroughbred horse populations
Source: PLoS One. 2021 Feb 16;16(2):e0247123. doi: 10.1371/journal.pone.0247123 (PMC7886144; doi:10.1371/journal.pone.0247123)
Supplement: S1 Table — Due to unequal sample size, three iteractions, each of 20 randomly selected individuals was evaluated. Given is the mean and standard deviation (in parenthesis) of the replicates. (DOCX) [file pone.0247123.s001.docx]

**S1 Table.** Summary of the total number of runs of homozygosity by each size class. Due to unequal sample size, three iteractions, each of 20 randomly selected individuals was evaluated. Given is the mean and standard deviation of the replicates.

| ROH size (Mb) | Kurdish | Persian Arabian | Thoroughbred |
| --- | --- | --- | --- |
| 0-6 | 23321.0 (138.7) | 22207.3 (140.7) | 15594.7 (129.9) |
| 6-12 | 26.0 (2.6) | 88.0 (3.5) | 304.7 (8.0) |
| 12-24 | 6.7 (0.6) | 41.3 (7.6) | 100.3 (11.9) |
| 24-48 | 0.0 (0) | 11.0 (4.4) | 18.0 (1.0) |
| >48 | 0.0 (0) | 1.3 (1.2) | 0.3 (0.6) |
