# Supplementary material for: Genomic comparisons of Persian Kurdish, Persian Arabian and American Thoroughbred horse populations
Source: PLoS One. 2021 Feb 16;16(2):e0247123. doi: 10.1371/journal.pone.0247123 (PMC7886144; doi:10.1371/journal.pone.0247123)

**S2 Table.** Results on the comparisons of K=1 to 5 tested by STRUCTURE Harvester. The highlighted row belongs to the K value (=2) that maximizes Delta K per the Evanno method of determining the best fit for the data.


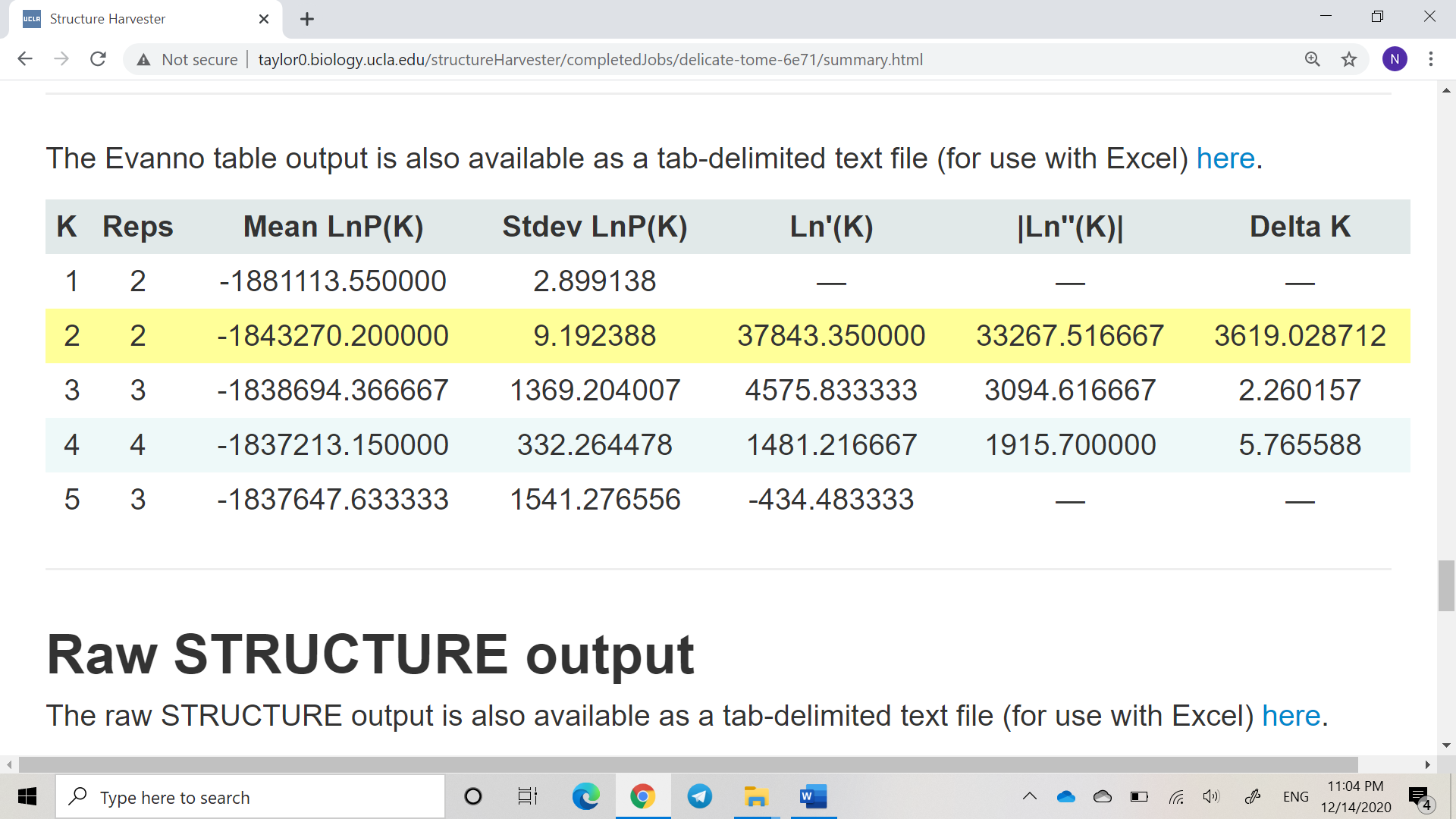

Supplement: S2 Table — The highlighted row belongs to the K value (= 2) that maximizes Delta K per the Evanno method of determining the best fit for the data. (DOCX) [file pone.0247123.s002.docx]
