# Supplementary material for: Genomic comparisons of Persian Kurdish, Persian Arabian and American Thoroughbred horse populations
Source: PLoS One. 2021 Feb 16;16(2):e0247123. doi: 10.1371/journal.pone.0247123 (PMC7886144; doi:10.1371/journal.pone.0247123)
Supplement: S3 Table — (DOCX) [file pone.0247123.s003.docx]

**S3 Table.** Average proportion of membership of each pre-defined population in each of the 3 clusters at K=3.

| Population | Cluster 1 | Cluster 2 | Cluster 3 |
| --- | --- | --- | --- |
| Kurdish | 0.042 | 0.942 | 0.016 |
| Persian Arabian | 0.519 | 0.463 | 0.017 |
| Thoroughbred | 0.023 | 0.023 | 0.953 |
