# Supplementary material for: Genomic comparisons of Persian Kurdish, Persian Arabian and American Thoroughbred horse populations
Source: PLoS One. 2021 Feb 16;16(2):e0247123. doi: 10.1371/journal.pone.0247123 (PMC7886144; doi:10.1371/journal.pone.0247123)
Supplement: S4 Table — (DOCX) [file pone.0247123.s004.docx]

**S4 Table.** Average proportion of membership of each pre-defined population (excluding the Thoroughbred samples from the dataset) in each of the 2 clusters at K=2.

| Population | Cluster 1 | Cluster 2 |
| --- | --- | --- |
| Kurdish | 0.969 | 0.031 |
| Persian Arabian | 0.457 | 0.543 |
